# A Key Node Ranking Method in Complex Networks by Integrating Semi-Local and Global Influence

**DOI:** 10.3390/e28101077

**Published:** 2026-09-30

**Authors:** Xiaohu Chen, Xiaoyang Liu, Jiaojiao Jiang, Chao Liu, Xin Wang

**Affiliations:** 1 School of Computer Science and Engineering, Chongqing University of Technology, Chongqing 400054, China; chenxiaohu@stu.cqut.edu.cn (X.C.); lxy3103@cqut.edu.cn (X.L.); 20140058@cqut.edu.cn (C.L.); 2 School of Computer Science and Engineering, University of New South Wales (UNSW), Sydney, NSW 2052, Australia; jiaojiao.jiang@unsw.edu.au; 3 School of Journalism and Communication, Chongqing University, Chongqing 400044, China

**Keywords:** complex network, critical node identification, semi-local influence, global influence, structural information, K-shell decomposition

## Abstract

The existing key node identification methods in complex networks rely on local topological information or global structural features, which are difficult to fully integrate multi-order local structural information and global propagation characteristics, resulting in the limitation of the accuracy and discrimination ability of node influence evaluation. In this paper, a key node ranking method combining semi-local and global influence, named SGIR, is proposed. Firstly, in the semi-local influence calculation stage, the second-order neighborhood, the third-order neighborhood, and the triangular closed structure of the node are used to construct the structural correlation matrix, and the structural energy migration mechanism is combined to quantify the local structural influence of the node, so as to enhance the expression ability of the semi-local structural features of the node. Secondly, in the global influence calculation stage, based on K-shell decomposition, a distance attenuation mechanism is introduced to weighted aggregate the influence of core nodes within the limited propagation range, and an adaptive propagation radius is designed by combining the average shortest path length of the network to effectively describe the global propagation ability of nodes. The semi-local influence and the global influence are fused to obtain the comprehensive influence ranking of nodes. Finally, comparative experiments are carried out on nine real complex networks from different fields such as communication, PowerGrid, and biological and social networks. The experimental results show that the proposed SGIR method achieves the best Kendall’s $\tau_{b}$ on most data sets, and produces a highly discriminative ranking, which verifies the rationality, effectiveness, and stability of the proposed method and provides an effective solution for the identification of critical nodes in complex networks that takes into account both semi-local structural information and global propagation ability.

## 1. Introduction

In the digital age, complex networks have become an important tool for describing and understanding complex systems in the real world. Whether it is social networks, electric power networks, transportation networks, biological networks, or infectious disease transmission networks, the analysis of the structure and function of complex networks has penetrated deeply into many fields of natural science and social engineering [1]. The essence of critical node identification is to rank the importance of all nodes in the network, aiming to find those nodes that have significant influence on the structure and function of the network. The research on this problem has a wide range of applications, covering public opinion control, marketing, power system protection, biological target discovery, and other aspects [2,3]. For example, in social networks, a small number of highly influential individuals can often guide public opinion or accelerate information dissemination [4]. In power networks, failures of key hub nodes may trigger large-scale cascading failures [5]. In an infectious disease network, timely identification and isolation of super spitters is crucial to contain the spread of an epidemic [6]. In addition, in medical diagnosis, researchers use the features extracted from chest CT scans collected by different devices to construct COVID-19 graphs and distinguish COVID-19 pneumonia cases from normal controls through graph convolutional networks [7]. In the field of equipment engineering, a state assessment method for complex task network models has been proposed to evaluate the available capabilities of avionics systems for each task in incomplete states [8]. Therefore, how to accurately identify the key nodes in complex networks has become one of the core topics in the field of network science. Because of this, how to accurately identify key nodes in complex networks has become one of the core topics in the field of network science.

In recent years, researchers have proposed a large number of node importance evaluation methods based on network topology for the identification of critical nodes in complex networks. According to the range of structural information utilized, the existing methods can be mainly divided into local centrality methods, global centrality methods, and local-global fusion centrality methods. The local centrality method mainly measures the influence of nodes by the topological characteristics of the node itself and its neighborhood, which has the advantages of high computational efficiency and easy expansion and is suitable for large-scale complex network scenarios. Typical methods include Degree Centrality (DC) [9], Neighborhood Degree (ND) [10], and Semi-Local Centrality (SLC) [11]. The degree centrality measures the importance of nodes by the number of direct connections between nodes. The sum of neighbor degrees further considers the influence of adjacent nodes, and the semi-local centrality extends the evaluation scope by introducing second-order neighborhood information. However, these methods mainly rely on local topological features in a limited range, ignoring the structural position of nodes in the whole network and the ability of cross-region propagation, so there are certain limitations in identifying nodes with similar local structures but obvious differences in global influence. In order to overcome the problem that the local method cannot describe the overall structure information of the network, the global centrality method further uses the overall topological characteristics of the network to evaluate the importance of nodes. Representative methods include Betweenness Centrality (BC) [12], Eigenvector Centrality (EC) [13], and K-shell decomposition method [14]. The betweenness centrality measures the bridging role of a node by the degree of its participation in the shortest path, the eigenvector centrality recursively evaluates the influence of a node by the importance of its neighbor nodes, and the K-shell decomposition reveals the position of the node in the overall structure by the hierarchical division of the network core. Although global methods can effectively capture the network embedding characteristics of nodes, they usually rely on the overall topological relationship. For nodes in the same core layer or with similar global index values, it is difficult to further distinguish their local structural differences and propagation potentials. In view of the shortcomings of local methods and global methods, in recent years, researchers have begun to explore the key node identification method by fusing multi-scale structural information. These methods improve the accuracy of node influence evaluation by combining local neighborhood features and global network information. For example, Gravity Model (GM) [15] draws on the idea of gravity in physics and combines node attributes with the distance between nodes to describe the propagation effect. The Local and Global Centrality (LGC) [16] method integrates the neighborhood information of nodes and the global structure of the network. Improved Local and Global Centrality (ILGC) [17] further optimizes the measurement method of local contribution and global influence. More recently, several studies have further advanced local-global fusion strategies. Mukhtar et al. [18] proposed a Hybrid Global Structure Model (H-GSM) that integrates local and global topological information to identify influential nodes with improved computational efficiency and discrimination ability. Zhang et al. [19] developed a semi-local centrality measure that integrates multidimensional factors, including neighborhood degree, clustering, and distance information, to enhance the identification of influential nodes. Pan et al. [20] designed a semi-local centrality measure based on an augmented graph and average shortest path theory, which redefines the semi-local structure to better capture large-scale network propagation characteristics. Liu et al. [21] combined a gravity box-covering model with effective distance to identify key nodes, further demonstrating the value of integrating propagation-distance information with structural attributes. Although these methods have improved the performance of key node identification by combining multi-order local features with global structural information to varying degrees, most of them still rely on relatively low-order local descriptors and do not fully exploit high-order structures such as triangular closures, nor do they explicitly model the distance-dependent attenuation of core-node influence under an adaptively determined propagation radius, which remains a key challenge for further research.

In fact, the influence of nodes in complex networks is not only determined by a single scale structure but is formed by the joint action of local embedding features and global propagation positions. On the one hand, the connection pattern between a node and its multi-order neighborhood nodes and the local closed structure can reflect the structural advantages of nodes in the local network. On the other hand, the location of a node in the core area and the distance relationship between the node and the core node determine its potential influence range in the process of network propagation. Therefore, it is easy to ignore the structural differences in the neighborhood of nodes only by relying on traditional local indicators, and it is difficult to distinguish the influence between nodes with similar core positions by simply using global indicators. These two types of structural information are logically complementary but not subsumable. The semi-local structural features mainly describe the strength and density of nodes embedded in their immediate multi-order neighborhood, reflecting their intrinsic local propagation potential. The global propagation capability, however, determines the extent to which this local potential can actually be extended and realized throughout the network. The joint modeling of the two can obtain a more complete node influence.

In order to solve the above problems, this paper proposes a novel ranking method of critical nodes in complex networks based on semi-local and global influence, named SGIR. Firstly, in the semi-local influence calculation stage, the structural correlation matrix is constructed by fusing the second-order neighborhood, the third-order neighborhood, and the triangular structure information of the node, and the local influence of the node is quantified by combining the structural energy migration mechanism, so as to describe the embedding degree and correlation strength of the node in the local structure more comprehensively. Then, in the global influence calculation stage, based on K-shell decomposition, a distance attenuation mechanism is introduced to weighted aggregate the influence of core nodes within a finite radius, and the propagation radius is adaptively determined according to the average shortest path length of the network, so that the global influence can not only reflect the core structure of the network but also take into account the propagation distance factor. Finally, the semi-local influence and the global influence were fused to obtain the comprehensive influence ranking of nodes. Compared with the existing hybrid centrality methods such as LGC and ILGC, the LGC and ILGC methods mainly rely on neighborhood aggregation based on the first or second order degree in the local part and rely on relatively static core-level information in the global part. The core conceptual advantages of SGIR are reflected in two aspects: first, by embedding the second-order neighborhood, third-order neighborhood, and triangle closure information into a unified structural correlation matrix, and quantifying it through the structural energy transfer mechanism, SGIR captures the high-order local embedding patterns that go beyond the degree-based descriptors used by LGC and ILGC. Second, instead of directly summing or averaging the core-level metrics as LGC and ILGC do, SGIR introduces distance-decay aggregation of the influence of core nodes within an adaptively determined propagation radius, thus explicitly modeling how the global propagation advantage decays with its distance from the network core.

The main contributions of this paper are as follows:

(1) A key node identification method, SGIR, is proposed by fusing semi-local and global topology information, which unifies the local structural characteristics of nodes and the global propagation ability of nodes and improves the accuracy of node influence evaluation.
(2) A structural correlation matrix combining the second-order neighborhood, the third-order neighborhood, and the triangular structure is constructed, and a structural energy shift mechanism is designed to effectively enhance the expression ability of semi-local structural information.
(3) A distance attenuation global influence model based on K-shell is proposed, and an adaptive radius strategy is adopted to improve the ability of describing global influence while ensuring computational efficiency.
(4) A large number of experiments were carried out on 9 real complex networks. The experimental results show that the proposed SGIR method is superior to several classical methods in terms of Kendall’s $\tau_{b}$, recognition rate, CCDF, Top 10 key node identification, and other evaluation indicators, which verifies the effectiveness and stability of the proposed SGIR method.

## 2. Related Work

The identification of key nodes in complex networks is one of the core problems in the field of network science, which aims to mine the nodes that have important influence on the structure and function of the network from the network structure. Over the years, researchers have proposed a large number of methods from different perspectives. According to the different topological information used, these methods can be roughly divided into three categories: local centrality methods, global centrality methods, and hybrid centrality methods.

### 2.1. Key Node Identification Method Based on Local Centrality

The local centrality method uses the topological information of the node itself and its neighborhood to evaluate the importance of the node, which has low computational complexity and good scalability for large-scale networks. DC proposed by Freeman [9] is the most representative local centrality index. However, this method is difficult to effectively distinguish the nodes with the same degree value but in different network locations. Inspired by the idea of H-index in academic evaluation, Hirsch [22] proposed the H-Index (HI) method to evaluate the influence of nodes by comprehensively considering the distribution of neighbor node degree values. With the deepening of research, scholars have incorporated the local structural relationships and interaction patterns between nodes into the evaluation process. Lu et al. [23] proposed the ESCRM method to comprehensively evaluate the importance of nodes by combining the structural similarity and local interaction between nodes. Wang et al. [24] further considered the local clustering characteristics of nodes and structural hole information and proposed the SNC method. In order to expand the scope of local information perception while ensuring computational efficiency, Chen et al. [11] proposed the SLC method. The proposed method utilizes both the first-order and second-order neighborhood information of nodes. Local centrality methods have evolved from a single neighborhood index to multi-order neighborhood fusion, but they are still difficult to capture the global position and cross-region propagation ability of nodes because they only use the information near the node.

### 2.2. Key Node Identification Method Based on Global Information

The global centrality method uses the overall topology of the network to evaluate the importance of nodes, which can provide a broader spread ability and structural role perspective than the local method. BC [12] proposed by Freeman measures the importance of nodes by counting the number of nodes participating in the shortest path of the network. However, BC has high time complexity, and then Bonacich [13] proposed EC, which believes that the importance of a node depends not only on its own connection number but also on the influence of its neighbors. On the basis of EC, Page [25] proposed the PageRank algorithm, which introduced the idea of random walk into the evaluation of node importance, but its performance was affected by parameters such as the damping factor. Therefore, Lü et al. [26] proposed the LeaderRank (LR) algorithm to avoid the parameter setting process by introducing ground nodes to build a strongly connected network. The network core structure also affects the propagation: the K-shell decomposition proposed by Kitsak et al. [14] divides the network core level by stripping the low-degree nodes layer by layer and believes that the nodes in the high-core level have stronger propagation potential. However, it is only evaluated based on the core layer, and it is difficult to distinguish the nodes with the same K-shell value. To this end, Liu et al. [27] optimized the core layer division by removing redundant edges, and Wang et al. [28] introduced an iterative influence factor to fuse neighbor influence to achieve a finer sorting. In recent years, some studies have further extended K-shell by combining node distance, propagation path, and global structure information, which effectively improves the recognition performance of the global centrality method.

### 2.3. Key Node Identification Method Based on Mixed Centrality

In recent years, in order to make up for the lack of local and global centrality, researchers have begun to explore the key node identification method that integrates local topology and global structure, which improves the accuracy of node influence evaluation to a certain extent. Bae et al. [16] proposed the LGC method, which combined the local connection characteristics of nodes with the global structure information of the network to realize the comprehensive evaluation of the local influence and the overall propagation ability of nodes. However, LGC does not consider the connection relationship between neighborhoods and higher-order local structures. In order to solve the local knot problem of LGC, Wang et al. [17] proposed an improved local and global centrality method, ILGC. The method further introduces the degree information and K-shell value of neighbor nodes, comprehensively considers the node’s own characteristics and the contribution of neighbor nodes in the local influence calculation, and uses the network core level information to enhance the global influence evaluation ability, so as to improve the identification effect of key nodes. At the same time, Liu et al. [29] proposed the KSvoterank method, which combined K-shell decomposition with an enhanced voting mechanism and dynamically updated the voting ability of nodes by considering the contribution of direct neighbors and indirect neighbors. This method effectively disperses the identified key nodes, alleviates the redundant aggregation effect when selecting nodes only according to the centrality score, and shows good performance in terms of propagation range and robustness. However, the existing methods still have shortcomings. On the one hand, local influence modeling usually relies on low-order topological features such as node degree and neighbor degree and fails to make full use of high-order structural information such as third-order neighborhood and triangle closure. On the other hand, the global influence evaluation is mostly based on the core hierarchy or the overall topological relationship of nodes, which is difficult to fully reflect the attenuation characteristics of the influence of core nodes under different propagation distances. Therefore, this paper proposes a new method based on semi-local and global influence to rank critical nodes in complex networks, named SGIR. In the phase of semi-local influence calculation, the multi-order neighborhood relationship and triangle closure structure information are introduced to enhance the local structure expression ability of nodes. At the same time, in the global influence calculation stage, the position and distance attenuation mechanism of the core node are combined to characterize the global influence contribution under different propagation ranges.

It is worth noting that, although GNN-based methods have recently been applied to key node identification with competitive accuracy, they typically require large-scale training data, incur high computational and memory overhead, and offer limited interpretability. In contrast, the topological methods reviewed above require no training, have low computational complexity and good interpretability, and are thus more advantageous for engineering deployment in large-scale or real-time scenarios.

## 3. Preliminaries

Let G(V,E) be a network graph where the set of nodes is denoted by $V=\{v_{1},v_{2},\ldots ,v_{n}\}$, the set of edges is defined as $E\subseteq \{\{v_{i},v_{j}\}\;|v_{i},v_{j}\in V,i\neq j\}$, For any edge $\;e_{ij}=\;\left\{v_{i},v_{j}\right\}$ it represents a connection between node $v_{i}$ and $v_{j}$. $\left|V\right|=n$ denotes the number of nodes, and $\left|E\right|=m$ denotes the number of edges. The network graph can be represented using the adjacency matrix A of n×n, where the matrix element $a_{ij}$ takes the value 1 to indicate the existence of an edge connection between node i and node j, and the value 0 to indicate no connection.

### 3.1. Degree Centrality

DC [9] describes the number of directly connected neighbors of a node as the most direct and straightforward measure of the importance of a node. The formula is as follows:

(1) $$DC(i)=\frac{k_{i}}{\left(n-1\right)},$$

where $k_{i}$ represents the degree of node i and n represents the total number of nodes in the network.

### 3.2. Betweenness Centrality

BC [12] quantifies the extent to which a node lies on the shortest path between other nodes, highlighting its role in connecting different parts of the network. It is defined as follows:

(2) $$BC(i)=\sum_{v,u\in N}\frac{\sigma (v,u|i)}{\sigma (v,u)},$$

where σ(v,u) represents the number of shortest paths between nodes v and u, and σ(v,u|i) represents the number of shortest paths between nodes v and u that pass-through node i.

### 3.3. Neighborhood Degree

ND [10] is a local centrality measure that takes into account not only the degree of the node itself but also the influence of its neighbors. It can distinguish those nodes with low potential influence but connects multiple higher nodes, which may play a key role in information transfer. It is defined as follows:

(3) $$ND(i)=\sum_{j\in \Gamma (i)}k_{j},$$

where $k_{j}$ represents the degree of node j and Γ(i) is the set of neighborhoods of node i.

### 3.4. Semi-Local Centrality

SLC [11] is a node importance evaluation method combining local and neighborhood information, which is calculated based on the degree of a node and the degree of its nearest neighbors. It is defined as follows:

(4) $$SLC(v)=\sum_{w\in \Gamma (v)}Q(w),$$

where Γ(v) represents the set of first-order neighbors of the node v, $Q(w)=\sum_{u\in \Gamma (w)}k_{u}$ is the second-order neighbor information contribution of node w. Γ(w) be the set of first-order neighbors of node w and $k_{u}$ be the degree of node u.

### 3.5. K-Shell

K-shell (KS) decomposition [14] is a key node identification method based on the position level of nodes in the network. The network is divided into different levels by iterative peeling of nodes whose degree is less than or equal to k, and the K-shell value of a node reflects its core position in the whole network.

### 3.6. Gravity Model

GM [15] measures the propagation influence between each pair of nodes based on the law of gravity. In this model, the degree of a node is regarded as the quality, and the shortest path between nodes is regarded as the mutual attraction distance between nodes. The influence of a node is given by the following formula:

(5) $$GM(i)=\sum_{j\neq i}\frac{k_{i}\times k_{j}}{d_{ij}^{2}},$$

where $d_{ij}$ represents the shortest path length between nodes i and j. The influence of a node depends not only on its own influence, but also on the influence of its neighboring nodes.

### 3.7. Local and Global Centrality

LGC [16] is a key node identification method that considers both local influence and global influence of nodes. It is calculated based on the degree of nodes, the number of nodes in the whole network, and the shortest path between nodes. It is defined as follows:

(6) $$LGC(i)=\frac{k_{i}}{n}\times \sum_{j\neq i}\frac{\sqrt{k_{j}+\alpha }}{d_{ij}},$$

where α is a tunable parameter, $k_{j}$ represents the degree of node j, and $d_{ij}$ represents the shortest path distance between node i and node j.

### 3.8. Improved Local and Global Centrality

ILGC [17] is a node influence identification method that combines local neighborhood degree information and global neighborhood kernel layer information and calculates the node propagation influence by aggregating the first-order and second-order neighbor information of the target node. It is defined as follows:

(7) $$ILGC(i)=\sum_{j\in \Gamma_{i}}k_{j}+\sum_{j\in \Gamma_{i}^{2}}\frac{ks(j)}{d_{ij}},$$

where $\Gamma_{i}$ denotes the set of first-order neighbors of node i, and $k_{j}$ denotes the degree of node j. Let $\Gamma_{i}^{2}$ denote the set of first-order and second-order neighbors of node i, let ks(j) denote the K-shell value of node j, and $d_{ij}$ represents the shortest path distance between node i and node j.

## 4. Proposed Method

**Figure 1:**
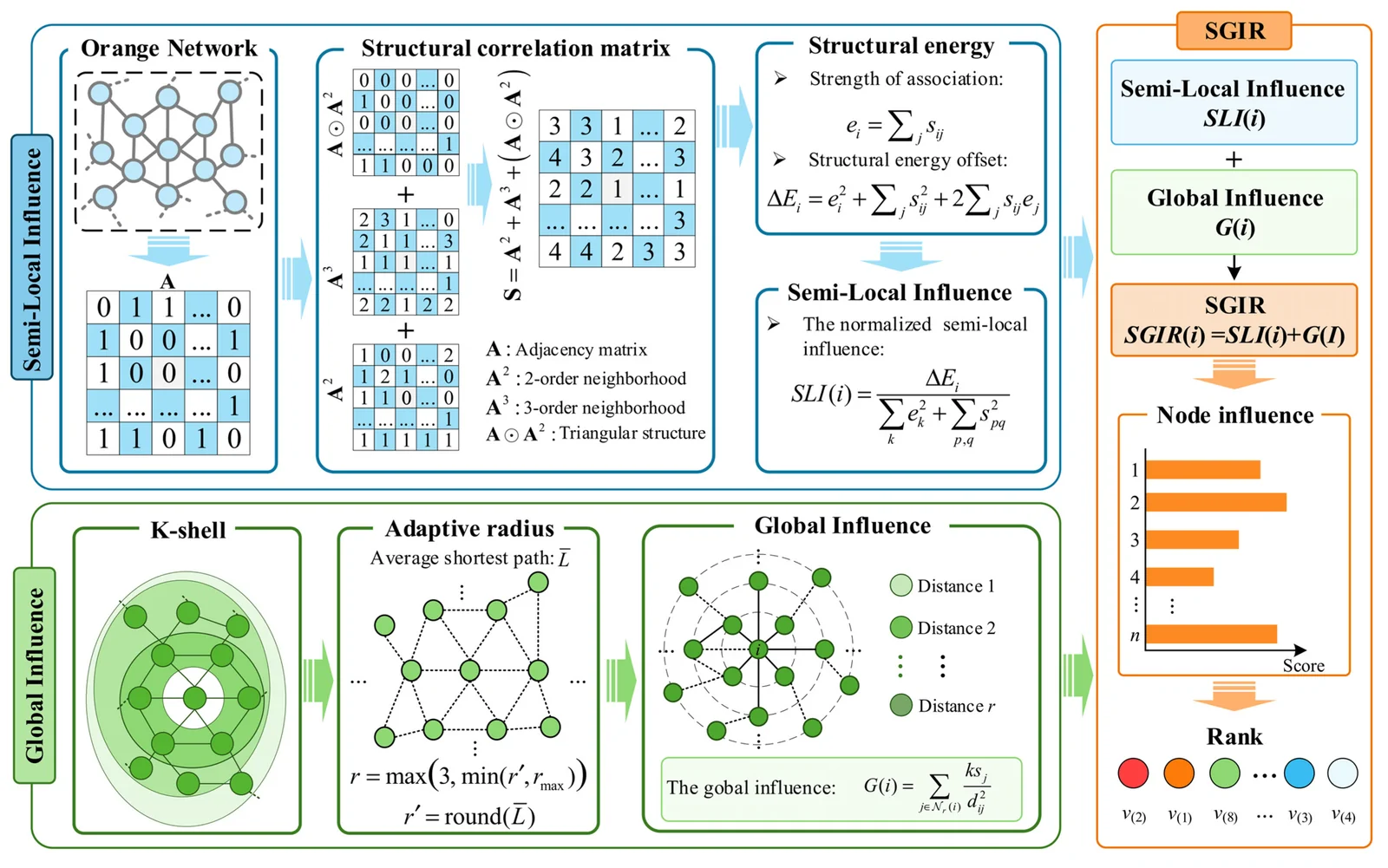
Framework diagram of the SGIR method.

The framework of the SGIR method is shown in Figure 1, which mainly consists of three parts: semi-local influence calculation, global influence calculation, and comprehensive influence fusion. In the semi-local influence stage, the correlation matrix combining the second-order and third-order neighborhood information, and the triangular structure information is constructed, and then the local embedding strength of the node is quantified by the structural energy shift mechanism and normalized to obtain the semi-local influence. In the global influence stage, the K-shell decomposition is performed to obtain the core level of nodes, and then the propagation radius is adaptively determined according to the average shortest path of the network. The distance attenuation is used to aggregate the gravitational contribution of core nodes in a limited range to obtain the global influence. Finally, the two are linearly fused to generate the comprehensive score, and the ranking of key nodes is output in descending order.

### 4.1. Semi-Local Influence Calculation

The local influence of a node is closely related to its neighborhood structure. Most existing studies use the second-order neighborhood to measure the structural similarity between nodes, but these methods are limited to local second-order information, and it is difficult to effectively describe high-order structural features, such as local density embodied by triangular closed structure. Therefore, the second-order neighborhood, the third-order neighborhood, and the triangular structure information are taken into account when calculating the semi-local influence. In order to comprehensively describe the multi-order structural correlation between nodes, the structural correlation matrix S is defined as follows:

(8) $$S=A^{2}+A^{3}+\left(A⊙A^{2}\right),$$

where A is the adjacency matrix, $A^{2}$ reflects the number of paths in the second-order neighborhood of the node, $A^{3}$ reflects the number of paths in the third-order neighborhood of the node, and $A⊙A^{2}$ is expressed as the enhanced encoding of the triangular structure, highlighting the mutual reinforcement between the node pairs with close local clustering.

After obtaining the correlation matrix S, we first aggregate the multi-order correlation information in the row vector corresponding to node i to define the structural correlation strength of the node:

(9) $$e_{i}=\sum_{j}s_{ij},$$

where $e_{i}$ measures the overall degree of structural association between node i and its higher-order neighborhood nodes. A larger value of $e_{i}$ indicates that the node has stronger local structural connection ability and plays a more important role in the local topology of the network. However, it is difficult to fully describe the distribution characteristics of association information in the neighborhood by only using the first-order aggregation results. Therefore, a structural description term based on the second moment is further introduced to describe the distribution characteristics of node association strength. Therefore, the node structure energy offset is defined as follows:

(10) $$\Delta E_{i}=e_{i}^{2}+\sum_{j}s_{ij}^{2}+2\sum_{j}s_{ij}e_{j},$$

where $e_{i}^{2}$ represents the square amplification of the total association strength $e_{i}$ of node i to enhance the basic weight of its own energy in the overall measurement. $\sum_{j}s_{ij}^{2}$ it describes the concentration degree of the correlation strength of a node on its neighborhood nodes, the larger the value is, the more concentrated the structural correlation of a node is on a few strong correlation neighbors; $2\sum_{j}s_{ij}e_{j}$ represents the coupling effect between the structural influence of nodes and neighboring nodes, so that the nodes connecting with strong structural contribution nodes can obtain higher evaluation. Therefore, $\Delta E_{i}$ considers the structural contribution of the node itself, the correlation distribution characteristics of the neighborhood, and the influence propagation relationship of the neighbor structure, and the multi-dimensional description of the local high-order structural characteristics of the node is realized.

This construction of multi-dimensional continuous values endows the structural energy shift with an intrinsic tie-breaking ability that is incomparable to purely local, integer-valued measures. The scores assigned to each node by metrics such as DC and KS are determined by only a small range of integer numbers. Therefore, nodes with the same degree or the same shell layer must receive the same score, which leads to the widespread ranking tie characteristic of these methods. Since the concentration and coupling terms are calculated from the real values, path counts and triangular weighting elements of the structural correlation matrix rather than from discrete counts, and they are combined with the squared amplitude term by nonlinear operations rather than simple sums, two nodes of the same degree or the same K-shell index. The distribution of the correlation strength in the higher-order neighborhood is usually different from the coupling mode, so their offset values are rarely exactly the same. This continuous sensitivity to the fine-grained distribution of neighborhood correlations enables structural energy shifts to eliminate parallelism in a way that discrete measures based on degrees or shell scores cannot.

In order to reduce the impact of network scale differences on the evaluation results of node importance, the overall structure energy of the network is used as the normalization benchmark, and the node structure energy offset is normalized to define semi-local influence:

(11) $$SLI(i)=\frac{\Delta E_{i}}{\sum_{k}e_{k}^{2}+\sum_{p,q}s_{pq}^{2}},$$

where $\sum_{k}e_{k}^{2}$ measures the overall magnitude of node self-correlation and $\sum_{p,q}s_{pq}^{2}$ measures the overall high-order correlation of the network. Together they serve as an energy-normalization baseline that reduces the effect of network size and structural density, so that SLI(i) is comparable across networks. A larger SLI(i) indicates a stronger contribution of node *i* to the local high-order structure.

### 4.2. Global Impact Calculation

Local structure information can effectively describe the connection characteristics of nodes in the neighborhood, but the information propagation process in complex networks is usually affected by the overall topology of the network. Therefore, it is difficult to fully evaluate the global propagation ability of nodes by only relying on local structural features. K-shell decomposition is a classical network core structure analysis method, which iteratively peels off low connectivity nodes, divides the network into different levels of core structures, and assigns the corresponding core value $ks_{i}$ to each node. A larger value of $ks_{i}$ indicates that the node is in a more central position in the network and has a stronger potential dissemination ability. However, the traditional K-shell method mainly focuses on the core level of the node, and ignores the structural differences between the nodes within the same core level. In fact, even if two nodes have the same K-shell value, their actual transmission ability may still be significantly different due to their different neighborhood node distribution, core node connection, and network distance relationship.

In order to solve the above problems, the idea of the gravity model was introduced; the core degree of nodes was regarded as the influence quality, and the shortest path distance between nodes was regarded as the propagation attenuation factor. According to the classical gravity model, the interaction strength between nodes usually increases with the enhancement of node attributes and decreases with the increase of distance. Therefore, the global influence of node j on node i is defined as follows:

(12) $$F_{ij}=\frac{ks_{j}}{d_{ij}^{2}},$$

where $ks_{j}$ represents the K-shell value of node j, which represents its influence ability in the core structure of the network. Considering that the influence of distant nodes in the network on the local propagation process is usually weak, in this paper, only the gravitational contribution of nodes in a limited range is calculated. For a node i, the global gravitational centrality within its radius r is defined as follows:

(13) $$G(i)=\sum_{j\in N_{r}(i)}\frac{ks_{j}}{d_{ij}^{2}},$$

where $N_{r}(i)$ represents the set of nodes centered at node i and not more distant than r. Compared with the traditional K-shell method, it not only pays attention to the attributes of the core layer of the node but also further considers the topological distance relationship between the node and the high core node, so that the node located in the center of the core area and connecting multiple high-level nodes can obtain higher evaluation.

The choice of the action radius r will directly affect the range of global information acquisition. If r is too small, nodes can only perceive limited neighborhood information, which easily leads to insufficient global structure information. If r is too large, a large number of weakly correlated nodes will be introduced, while increasing the computational complexity. Therefore, this paper proposes an adaptive radius selection strategy based on the average shortest path length of the network, so that the range of action can be dynamically adjusted according to the characteristics of different network structures. First, calculate the average shortest path length of the network:

(14) $$\bar{L}=\frac{1}{\left|V|(|V|-1\right)}\sum_{i,j\in V}d(i,j),$$

where V denotes the set of nodes. For non-connected networks, in this paper, the average shortest path length is calculated only on the largest connected subgraph to avoid the impact of isolated nodes on the network scale estimation. The initial propagation radius is determined based on the average path length:

(15) $$r^{\prime }=\mathrm{round}(\bar{L}),$$

where round(⋅) is the round function. Then, in order to avoid the influence of too small or too large propagation range on the stability of the index, the initial radius is constrained:

(16) $$r=\max(3,\;\min(r^{\prime },r_{\max})),$$

where $r_{\max}=5$ is the maximum radius of action, and the reason for setting the minimum radius to 3 is that for a highly dense small-scale network, a small radius may not cover enough structural information. However, limiting the maximum radius to 5 can reduce unnecessary computational overhead while ensuring the global information acquisition ability. Complex networks usually have obvious small-world characteristics, and the structural association between nodes will weaken rapidly after a certain path length. Therefore, limiting the search range can achieve a balance between information integrity and computational efficiency.

### 4.3. Total Influence

After obtaining the semi-local and global influence, the two are first subjected to min-max normalization and then combined to obtain the node’s comprehensive influence score:

(17) $$\bar{SLI}(i)=\frac{SLI(i)-SLI_{\min}}{SLI_{\max}-SLI_{\min}}$$

(18) $$\bar{G}(i)=\frac{G(i)-G_{\min}}{G_{\max}-G_{\min}}$$

where $SLI_{\max/\min},G_{\max/\min}$ are the maximum/minimum values over all nodes. After obtaining the normalized semi-local influence and global influence, the two are combined to obtain the comprehensive influence score of each node:

(19) $$SGIR(i)=\bar{SLI}(i)+\bar{G}(i),$$

where SGIR(i) represents the total influence of the target node. After obtaining the comprehensive influence score of each node, it is sorted in descending order, where a higher score indicates a more important node in the network. The sorted node list is the final key node ranking, which can be directly used for subsequent network analysis and application decision-making. The SGIR algorithm flow is given by Algorithm 1.
**Algorithm 1** SGIR**Input:** Network G(V,E);**Output:** Ranking list *Rank*;1 Construct the structural association matrix **S**;2 Compute the structural energy offset $\Delta E_{i}$;3 Normalize $\Delta E_{i}$ to obtain SLI(i);4 Perform K-shell decomposition;5 Compute the adaptive search radius *r*;6 **for** each node *i*
**do**:7   Compute global influence G(i);8 **end for**9 **for** each node *i*
**do**:10 Min–max normalize SLI(i) and G(i);11    $SGIR(i)\leftarrow \bar{SLI}(i)+\bar{G}(i)$;12 **end for**13 Sort Score in descending order;14 **Return**
*Rank*.

## 5. Experimental Analysis

### 5.1. Dataset

In order to evaluate the performance of the proposed algorithm, nine real networks from different fields such as communication, power grid, and biology are selected as test sets to evaluate the effectiveness of the proposed method. A brief description of these networks is as follows:

Sex [30]: A two-sided sexual activity online community in which the nodes are men (sex buyers) and women (sex sellers), and edges are established when men write posts indicating sexual activity with women.

PowerGrid [31]: A network containing information about the power grid in the western United States of America.

Router [32]: A network contains autonomous systems of the Internet that are interconnected.

pgp [33]: DataSet demonstrates a network of users named Pretty-Good-Privacy for secure data exchange algorithms.

Vidal [34]: Network for an initial version of the proteome fraction map for human binary protein–protein interactions.

GrQC [35]: Research collaboration network of authors in the field of general relativity and quantum cosmology, where nodes are authors and edges are established when two authors co-write a paper.

Hep [36]: Author Research Collaboration Network in High energy Physics, where nodes are authors and edges are established when two authors work together on a paper.

Stelzl [37]: Networks represent interacting protein pairs in humans.

Figeys [37]: Networks of interactions between human proteins from the first large-scale study of protein–protein interactions in human cells using mass spectrometer-based methods.

Table 1 summarizes the basic information of the networks in the dataset, *n* represents the number of nodes, m represents the number of edges, *<k>* represents the average node degree, *k*_max_ represents the maximum node degree, *C* represents the average clustering coefficient, *d* is the density of the network, and $\bar{L}$ is the average shortest path length.

### 5.2. Evaluation Metrics

#### 5.2.1. SIR Model

To measure the impact of the target node, the SIR model [38] is used to estimate the expected normalized spreading size of the target node, obtained by averaging the results over 1000 independent simulations, with the averaging explicitly incorporated into the formula below. The influence of the target node i is calculated as follows:

(20) $$F(i)=\frac{1}{N}\sum_{r=1}^{N}\frac{n_{r}^{i}}{n}$$

where N=1000 is the number of independent SIR simulations, $n_{r}^{i}$ denotes the number of infected nodes at the end of the  r−th independent run, and r is the total number of nodes. F(i) thus represents the expected proportion of infected nodes (i.e., the normalized spreading size) averaged over the N simulations. For the SIR-based reference ranking, each node in the network is successively independently selected as a unique initial infection seed, and the above SIR Propagation process is simulated 1000 times against this seed for a given infection probability. Then, the spreading influence of a node is obtained by averaging the number of infected nodes in these 1000 independent simulations. Subsequently, all nodes are sorted in descending order of average spread influence, that is, the SIR-based reference ranking is obtained. The infection probability varies in the range of $\beta /\beta_{\mathrm{th}}\in \left[1.0,1.9\right]$, where the threshold $\beta_{\mathrm{th}}$ is calculated as: $<k>/(<k^{2}>-<k>)$, <k> and $<k^{2}>$ denote the first and second moments of the network degree distribution, respectively. The lower bound $\beta /\beta_{\mathrm{th}}=1.0$ is the epidemic threshold itself, below which the epidemic dies out almost immediately and the spread scale is dominated by random noise. The upper bound $\beta /\beta_{\mathrm{th}}=1.8$ is chosen because a higher value will drive the network to saturation, so that the difference in propagation scale between nodes disappears and the sorting loses its distinguishing ability. This interval covers the transition region where the propagation result is still sensitive to the node structure position, so as to better reflect the difference of propagation influence between nodes.

#### 5.2.2. Kendall’s $\tau_{b}$ Correlation Coefficient

Kendall’s $\tau_{b}$ correlation coefficient [39] measures the ordinal agreement between the predicted ranking and the SIR-based reference ranking. It is defined as:

(21) $$\tau_{b}=\frac{P-Q}{\sqrt{\left(P+Q+T)(P+Q+U\right)}},$$

where P and Q denote the numbers of concordant and discordant node pairs, respectively; T is the number of node pairs tied only in the predicted ranking, and U is the number of node pairs tied only in the SIR-based reference ranking; a node pair that is tied in both rankings simultaneously is counted in neither T nor U. A larger value of $\tau_{b}$ indicates stronger agreement with the reference ranking.

#### 5.2.3. Monotonicity Index

Monotonicity index [40] evaluates the ability of a method to assign discriminative scores to nodes. It is defined as:

(22) $$MI={\left(1-\frac{\sum_{\alpha \in R}n_{\alpha }\left(n_{\alpha }-1\right)}{n\left(n-1\right)}\right)}^{2},$$

where R is the set of distinct ranking values, and $n_{\alpha }$ is the number of nodes assigned the value α. A value closer to 1 indicates fewer tied scores and stronger ranking discrimination.

#### 5.2.4. Recognition Rate $R_{p}$

The recognition rate [41] measures the degree of similarity between two node sets by calculating the proportion of the number of common nodes in the total size of the set. The higher the ratio is, the more consistent the two sets are in node composition, thus reflecting the representative or effectiveness of the selected set of nodes in the dissemination task. The recognition rate is defined as follows:

(23) $$R_{p}=\frac{\left|S_{p}\cap I_{p}\right|}{\left|I_{p}\right|},$$

where $R_{p}$ represents the ratio between sets $S_{p}$ and $I_{p}$.

#### 5.2.5. Complementary Cumulative Distribution Function

Complementary Cumulative Distribution Function (CCDF) [42,43] is an indicator for measuring the distinguishing ability of node influence ranking. The higher the value of CCDF, the stronger the distinguishing ability of the method for node influence. It is defined as follows:

(24) $$CCDF(l)=\frac{\left(n-\sum_{i=1}^{l}n_{i}\right)}{n},$$

where l represents the rank position (l=1 denotes the highest rank/most important), $n_{i}$ represents the number of nodes whose rank is exactly i, and $\sum_{i=1}^{l}n_{i}$ represents the total number of nodes whose rank is between 1 and l.

### 5.3. Performance Analysis Using Kendall’s $\tau_{b}$ Correlation Coefficient

To verify the effectiveness of the proposed SGIR method in the critical node identification task, Kendall’s $\tau_{b}$ correlation coefficient is used to measure the agreement between different node ranking methods and the SIR Model simulation results. A higher value of Kendall’s $\tau_{b}$ indicates that the node influence ranking generated by the proposed method is closer to the true spreading ability ranking, and its accuracy in identifying key spreading nodes is stronger.

**Figure 2:**
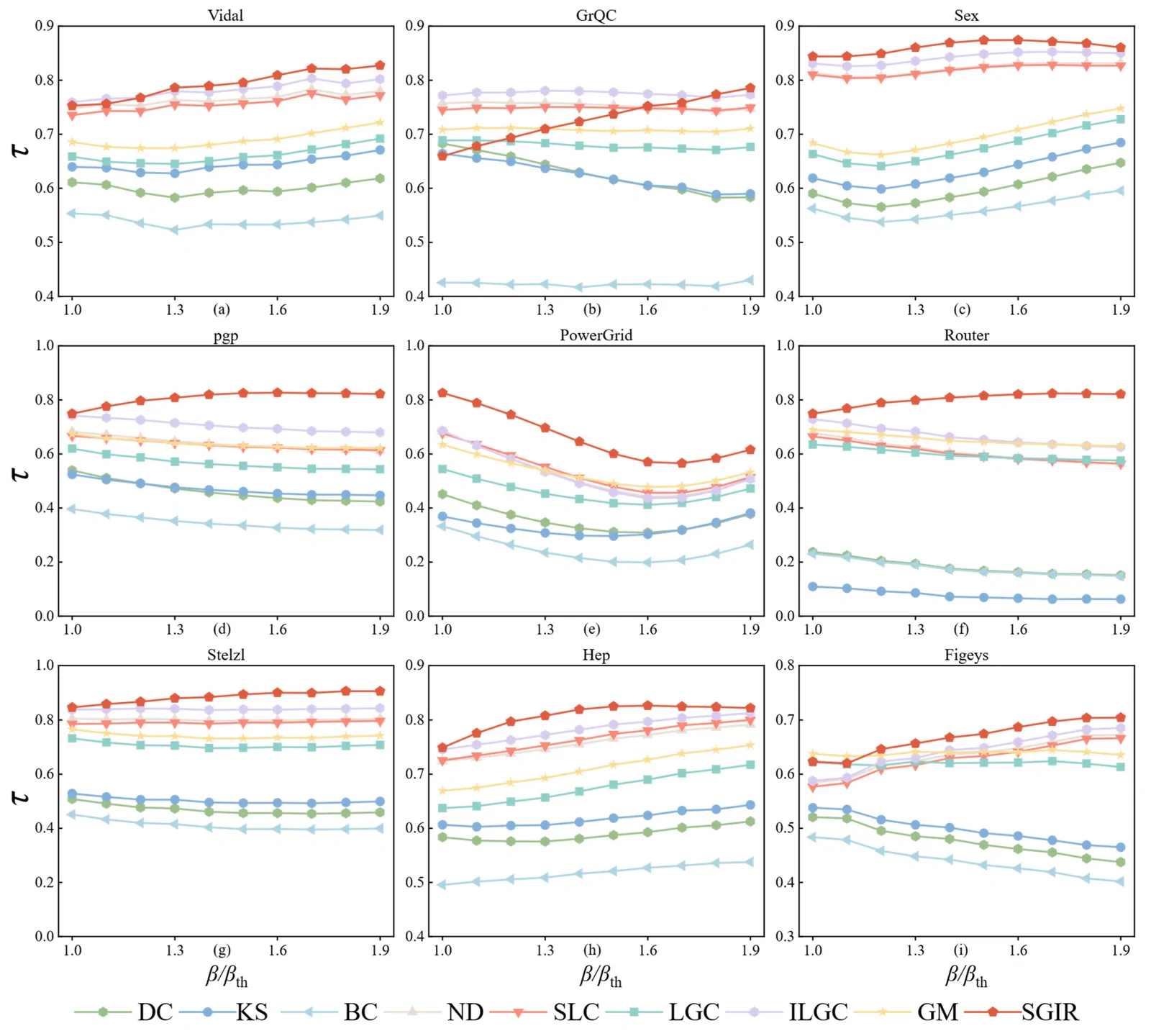
Node ranking capability. The ranking scores of DC, KS, BC, ND, SLC, GM, LGC, ILGC, and SGIR are compared with the ranking scores of simulated SIR Patterns on nine real networks under different infection rates β to obtain Kendall’s $\tau_{b}$ coefficient, where $\beta_{\mathrm{th}}$ is the expansion threshold. (**a**) Vidal; (**b**) GrQC; (**c**) Sex; (**d**) PGP; (**e**) PowerGrid; (**f**) Router; (**g**) Stelzl; (**h**) Hep; (**i**) Figsys.

In the experiment, SGIR was compared with DC, BC, SLC, ND, KS, GM, LGC, and ILGC methods and tested on nine real network datasets. The experimental results are shown in Figure 2. On the whole, SGIR obtains the highest Kendall’s $\tau_{b}$ value under most networks and different propagation probabilities, indicating that the proposed method can more accurately recover the real propagation influence ranking of nodes.

Specifically, in protein interaction networks such as Figeys, Vidal, and Hep, the $\tau_{b}$ value of SGIR is significantly better than that of other comparison methods. Such networks usually have a strong community structure, and it is difficult to accurately identify the nodes with strong cross-community propagation ability by only using local connection information. SGIR effectively captures the potential propagation role of nodes in the core area of the network by fusing high-order neighborhood structure and K-shell-based global influence information, so it shows better identification ability. In sparse networks such as PowerGrid and Router, SGIR similarly maintains a high Kendall’s $\tau_{b}$ value. Because there are few network nodes connected in sparse networks, it is easy to ignore the propagation contribution between distant nodes by simply relying on node degree or neighborhood information. SGIR introduces a distance attenuation mechanism to weight the influence of core nodes in different ranges, so that the global propagation ability of nodes can be more accurately described, and it has stronger adaptability and stability. The curve fluctuation of the PowerGrid network is more obvious; the main reason is that its network structure is sparse, and the degree distribution is uneven, which leads to low redundancy of propagation paths between nodes. The SIR Propagation results are more sensitive to initial seeds and random fluctuations. In addition, SGIR still maintains a high-ranking correlation in large-scale networks such as Sex and pgp, which indicates that the method is not only suitable for small-scale networks but also can effectively deal with complex large-scale network structures. Compared with LGC and ILGC methods that fuse local and global information, SGIR further introduces multi-order neighborhood correlation and structural energy features, which makes the node influence evaluation more comprehensive.

In summary, Kendall’s $\tau_{b}$ correlation coefficient experimental results show that the node rankings generated by SGIR have a higher consistency with the true propagation influence rankings obtained by the SIR Model. The results verify the effectiveness of SGIR in the key node identification task and also show that the joint modeling of semi-local influence and global influence can more accurately describe the node propagation ability in complex networks.

### 5.4. Discrimination Ability Experiment

**Figure 3:**
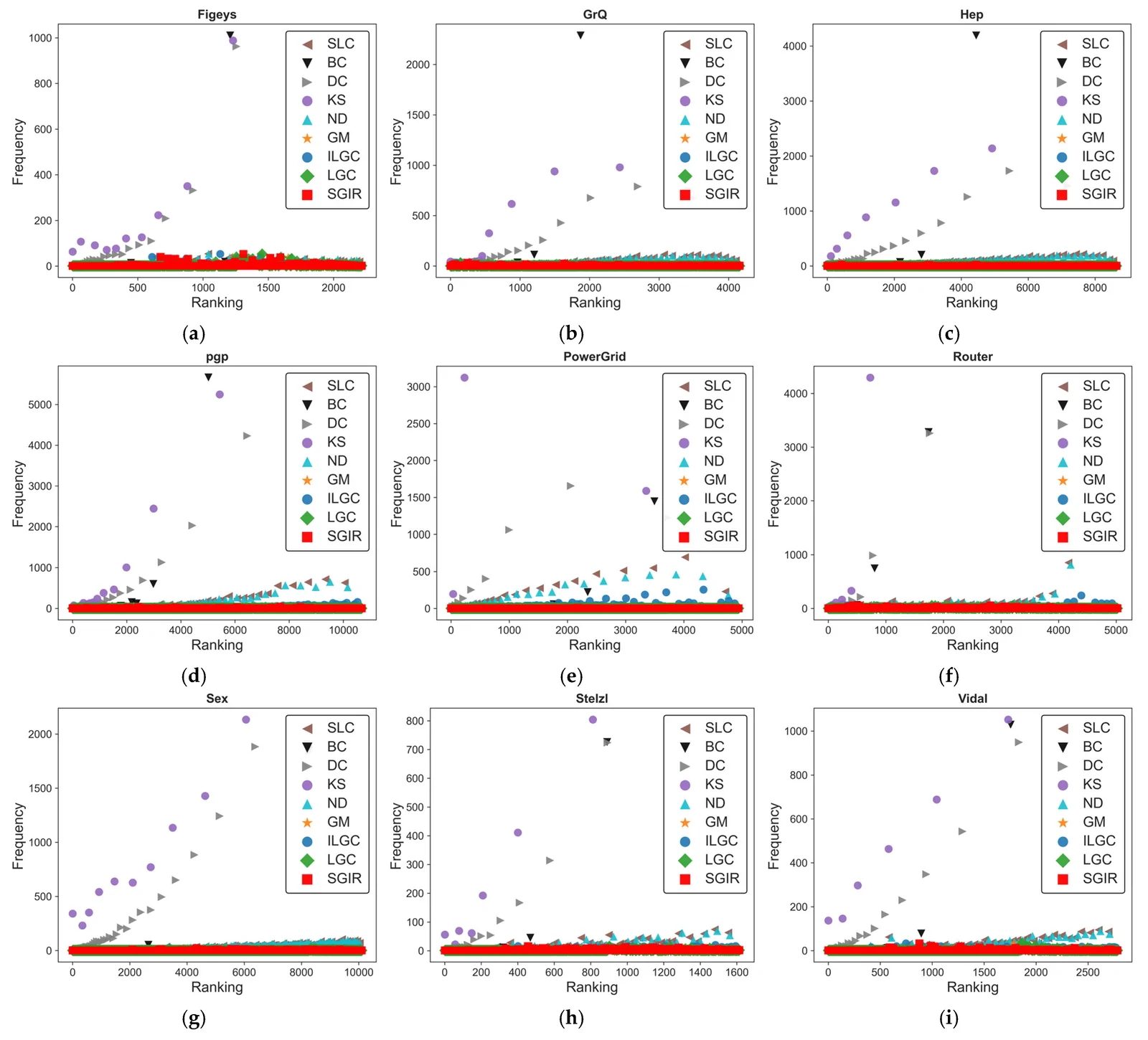
Frequency distribution of nine algorithms on nine networks. The *X*-axis shows the ranking of the nodes, and the *Y*-axis shows the frequency of occurrence of each ranking. (**a**) Figsys; (**b**) GrQC; (**c**) Hep; (**d**) pgp; (**e**) PowerGrid; (**f**) Router; (**g**) Sex; (**h**) Stelzl; (**i**) Vidal.

The discriminative power of centrality measure is important for accurately identifying critical nodes in complex networks. A centrality index with strong discrimination ability can assign more unique and reasonable importance scores to different nodes, reduce the ranking ambiguity problem caused by a large number of nodes obtaining the same score, and improve the reliability of node influence ranking. In order to evaluate the effectiveness of the proposed SGIR method in terms of node discrimination ability, we use SGIR and eight centrality metrics (including DC, BC, SLC, ND, KS, GM, LGC, and ILGC) to rank nodes in nine real networks, calculate the frequency of each rank, and visualize the frequency of each ranking derived from the proposed method and existing centrality measures. The experimental results are shown in Figure 3. As can be observed from the ranking frequency distribution in Figure 3, the node rankings produced by SGIR are shown in red, forming a distinct straight line at the bottom of almost all networks. This pattern shows that the ranking results generated by SGIR are more dispersed, effectively avoiding the situation where a large number of nodes share the same importance score. Thus, SGIR exhibits a strong ability to distinguish the vast majority of nodes in the network.

This advantage stems from the continuous, structure-sensitive way in which the structural energy offsets are constructed. Since their concentration and coupling terms are sensitive to the fine-grained distribution of correlation strength in the higher-order neighborhood of nodes, those nodes that cannot be distinguished under purely discrete measures such as degree or K-shell index will in most cases be assigned different scores by SGIR. While the scores of the traditional method are largely determined by this type of small-range dispersion, thus showing the tendency of ranking frequency concentration seen in Figure 3, SGIR consistently forms the scattered low-frequency line at the bottom of the distribution.

In order to quantitatively evaluate the discrimination ability of the proposed method, the monotonicity values of node ranking under the proposed method and the existing centrality metrics are calculated and compared. A higher monotonicity value indicates a more discriminative power of this centrality index. The results are shown in Table 2, where the optimal and suboptimal results in each column are marked with bold and single underscores, respectively. It can be observed that SGIR achieves high MI values in most networks and achieves optimal or near-optimal levels in several networks, indicating that it has more discriminative power than existing centrality measures.

**Figure 4:**
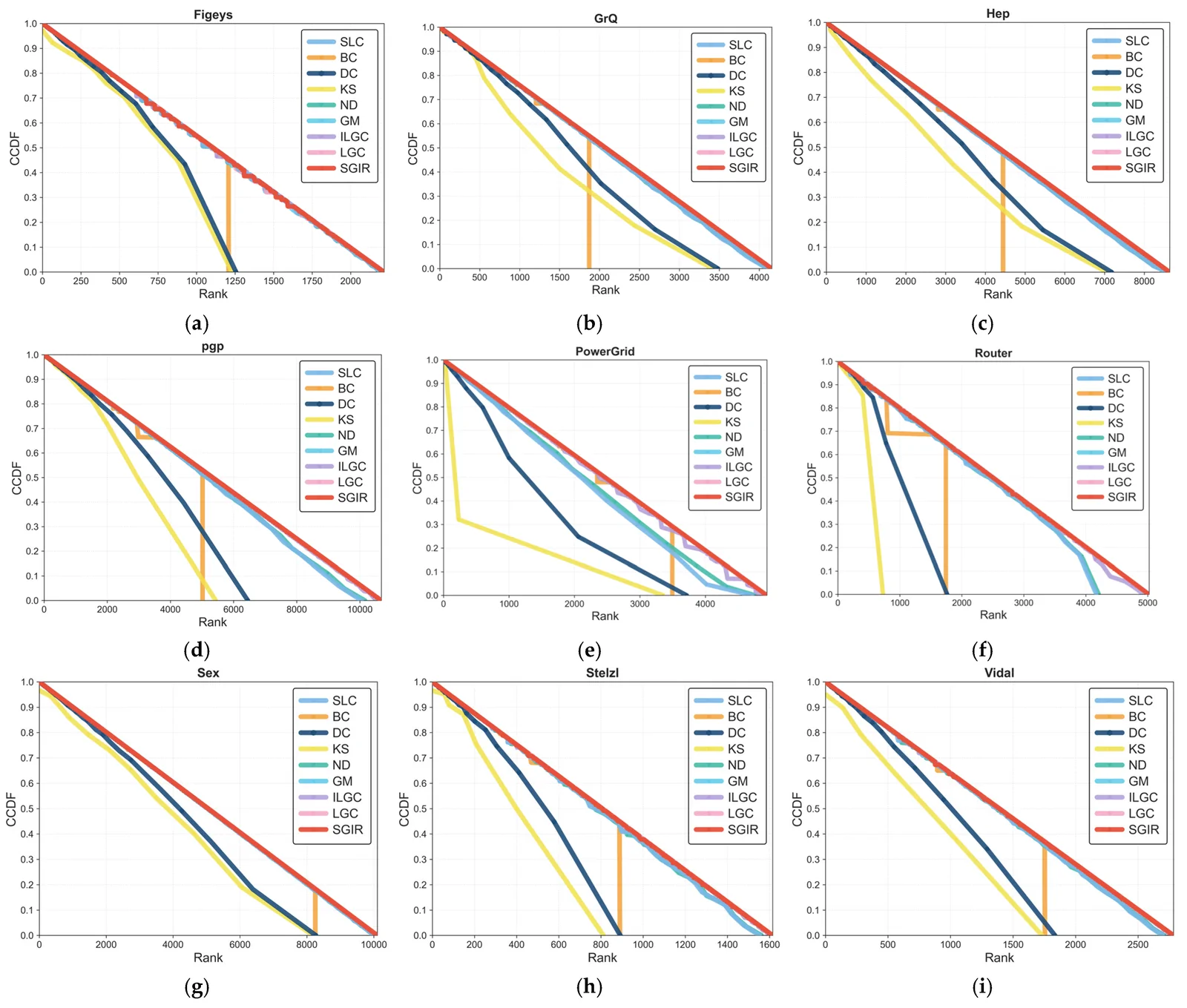
CCDF curves generated by SIGR and eight other methods on nine networks. (**a**) Figsys; (**b**) GrQC; (**c**) Hep; (**d**) pgp; (**e**) PowerGrid; (**f**) Router; (**g**) Sex; (**h**) Stelzl; (**i**) Vidal.

To further evaluate the node discrimination ability of different algorithms, CCDF is used for analysis. CCDF can reflect the distribution of the same ranking nodes in the node ranking results. When multiple nodes have the same rank, it is difficult to accurately distinguish their importance, and the algorithm with higher resolution can reduce the number of nodes with the same rank, so that the CCDF curve decreases more slowly. In this paper, CCDF experiments are carried out on nine real network datasets, and the SGIR method is compared with eight benchmark algorithms: SLC, BC, DC, KS, ND, GM, ILGC, and LGC. According to the node ranking results obtained by each algorithm, the corresponding CCDF values are calculated, and the CCDF curves of different methods are plotted, as shown in Figure 4.

From the experimental results, it can be seen that the CCDF curve of SGIR has the slowest decline speed in most networks, and the curve is smoother, which indicates that SGIR can effectively reduce the node ranking overlap phenomenon and has stronger node discrimination ability. In contrast, the curves of methods such as DC, KS, and BC decrease rapidly, which indicates that these methods are prone to producing nodes with the same ranking, resulting in reduced node identification ability.

Moreover, as the network size increases, the advantage of SGIR becomes more obvious. This is mainly due to the fact that SGIR considers both the semi-local structure information and the global influence information of nodes, which makes the node importance evaluation more refined. Therefore, SGIR shows high discriminative power in complex networks of different sizes and structures.

Although MI and CCDF are both used to characterize the discriminative power of node ranking methods, they characterize this property from different and complementary perspectives. MI is a single summary statistic that summarizes the overall proportion of pairs of nodes assigned different scores at the overall ranking level, thus providing a compact, network-level measure of how well a method avoids tie rankings. In contrast, CCDF is a distribution-level metric that shows how juxtapositions accumulate rank-by-rank along the entire ranking sequence, revealing where discriminative power degenerates.

### 5.5. Correlation Analysis

To systematically evaluate the difference between ILGC and SGIR in node influence identification performance, this study uses a ranking score correlation analysis method to conduct a comparative study. In the figure, the horizontal and vertical coordinates represent the normalized propagation influence scores of ILGC and SGIR on nodes, respectively. The diagonal reference lines marked in the figure provide an intuitive benchmark for performance comparison: the point set below the diagonal indicates that SGIR scores the corresponding node higher than the ILGC method, while the point set above the diagonal reflects that the ILGC method scores higher. By observing the distribution and color characteristics of the points on both sides of the diagonal, the performance differences of each method in identifying the spread influence of nodes can be effectively compared. The color of each point is determined by the propagation influence of the node obtained by simulating the SIR Model.

**Figure 5:**
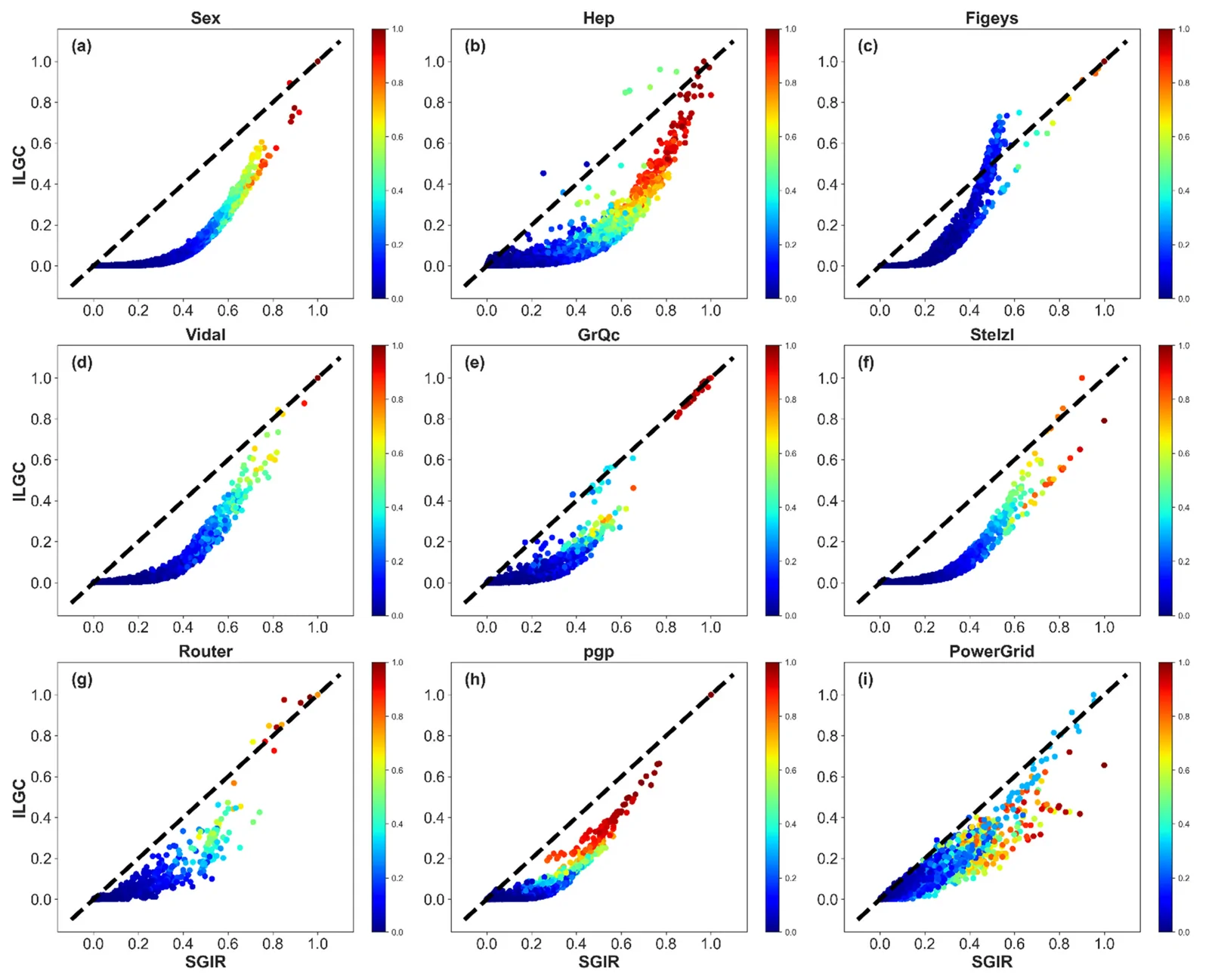
Correlation between normalized spread influence scores predicted by ILGC and SGIR on nine real networks. (**a**) Sex; (**b**) Hep; (**c**) Figeys; (**d**) Vidal; (**e**) GrQC; (**f**) Stelzl; (**g**) Router; (**h**) pgp; (**i**) PowerGrid.

It can be observed from Figure 5 that most of the red nodes are located below the diagonal. Compared with the ILGC method, the SGIR method can identify more important nodes and show a stronger ability to distinguish nodes with significant propagation influence.

### 5.6. Top 10 Nodes

In order to further verify the effectiveness of the SGIR method in identifying critical nodes, six real networks, Figeys, GrQC, pgp, PowerGrid, Router, and Vidal, are selected in this section. The Top 10 important nodes identified by SGIR and eight centrality methods such as DC, BC, SLC, ND, GM, LGC, and ILGC are compared and analyzed. The results are shown in Table 3, Table 4 and Table 5.

In Figeys network, SGIR shares 8 nodes with GM and LGC, 6 nodes with ILGC, and only 4 nodes with BC. This indicates that SGIR is consistent with LGC and ILGC methods, which also integrate multi-dimensional information in judging key nodes, while BC is biased to identify nodes that play the role of Bridges, and such nodes tend to have low degree values and limited actual propagation influence. In the GrQC network, SGIR coincides with GM in 7 cases, with LGC and ILGC in 6 cases each, with SLC and ND in 5 cases, and with BC in only 2 cases. In the pgp network, SGIR coincides with LGC in 7 cases, with DC and GM in 6 cases each, and again with BC in only 2 cases. It can be seen that in protein interaction networks and social networks, SGIR has high ranking consistency with GM and LGC methods based on global structure, while BC has the lowest overlap degree with SGIR in most networks, reflecting the limitation of betweenness centrality in identifying key nodes of propagation. In the sparse PowerGrid network, SGIR coincides with both SLC and ILGC in 7 cases, with GM and LGC in 4 cases each, and with DC in only 3 cases. This shows that in the power network with sparse connections, the degree centrality is difficult to use to effectively distinguish the node in the core or edge position of the network, and SGIR can more accurately identify the core node with real influence by introducing the global K-shell distance attenuation mechanism. In the Router network, the number of overlaps between SGIR and other methods is generally low, the highest is only 5, and the lowest is only 2. The structural characteristics of the Router network make the ranking results of different centrality indexes vary greatly, and the comprehensive ranking results of SGIR are more consistent with the SIR Simulation. In Vidal’s network, SGIR coincides with DC and GM in 7 cases each, LGC in 6 cases, and BC and ND in 3 cases each.

In general, SGIR has a high Top 10 overlap degree with GM, LGC, ILGC, and other methods that also integrate local and global information in most networks. At the same time, SGIR can integrate the advantages of multiple centrality indicators to identify the key node sets with more spreading influence.

**Table 3:** Top 10 important nodes for SGIR and other methods in Figyes and GrQC networks.

Rank	Figeys								GrQC							
	DC	BC	SLC	ND	GM	LGC	ILGC	SGIR	DC	BC	SLC	ND	GM	LGC	ILGC	SGIR
1	32	32	1174	32	32	32	32	32	101	1022	101	101	101	101	101	101
2	128	9	8	128	128	128	128	128	293	11	277	277	277	277	277	277
3	352	352	1079	1174	352	352	352	352	103	205	263	263	293	293	72	286
4	72	128	1152	352	72	72	72	72	277	53	72	72	103	103	77	72
5	123	72	1009	72	123	123	123	123	72	572	77	77	72	286	263	263
6	9	123	555	8	50	50	47	50	77	20	286	286	286	72	286	77
7	50	37	851	1079	158	9	8	158	294	145	103	103	77	77	293	103
8	158	89	32	1152	9	158	1174	7	286	184	294	293	263	263	103	293
9	89	309	47	47	7	7	1152	167	263	106	293	294	294	294	100	100
10	7	50	642	1009	226	89	1079	47	100	286	157	157	100	100	294	300

**Table 4:** Top 10 important nodes for SGIR and other methods in pgp and PowerGrid networks.

Rank	pgp								PowerGrid							
	DC	BC	SLC	ND	GM	LGC	ILGC	SGIR	DC	BC	SLC	ND	GM	LGC	ILGC	SGIR
1	9	9	9	9	9	9	9	9	3136	300	4516	4516	3136	197	4516	2707
2	79	659	79	79	79	79	79	79	197	661	4515	4515	197	3136	4522	4522
3	659	79	8	8	659	659	8	8	786	1683	4522	4522	660	2871	4515	4516
4	300	2570	143	143	8	8	143	143	3571	684	4555	4555	2871	660	4524	2760
5	8	300	19	19	143	143	19	659	4516	683	4524	4524	4516	2705	4546	4546
6	143	239	21	21	112	300	21	19	660	1425	4525	4525	2707	2699	4555	4524
7	112	829	16	16	19	112	16	16	2427	1749	4546	4546	2705	658	4525	4515
8	1508	235	659	659	21	19	659	21	2699	1459	4557	4557	2699	786	4557	1339
9	380	815	22	22	126	21	22	22	2871	509	4563	3136	658	2746	3136	3136
10	19	515	18	18	16	126	18	112	4045	3024	4549	4563	2746	2427	4542	2898

**Table 5:** Top 10 important nodes for SGIR and other methods in Router and Vidal networks.

Rank	Router								Vidal							
	DC	BC	SLC	ND	GM	LGC	ILGC	SGIR	DC	BC	SLC	ND	GM	LGC	ILGC	SGIR
1	25	2534	2534	304	25	25	2534	2534	128	367	146	128	128	128	128	128
2	152	1083	1083	2534	304	152	1083	1083	257	128	128	146	288	367	288	288
3	360	2723	304	1083	1081	360	304	1081	367	110	161	288	367	288	146	161
4	327	1081	1081	1081	360	304	1081	304	288	288	288	161	257	257	161	367
5	304	1300	2537	2537	152	1081	1300	1300	110	389	102	102	110	110	367	146
6	1082	2912	1300	1300	2534	1082	2537	1082	255	257	196	367	255	255	102	389
7	1081	2698	2536	1082	1082	327	1082	1299	329	286	160	196	329	329	196	257
8	9	413	1082	2536	1083	2534	192	2537	389	161	367	160	389	389	389	255
9	17	2716	192	192	1300	1083	2536	192	286	329	240	240	161	286	329	329
10	1083	1296	1299	1299	2537	1300	1299	2912	161	521	217	193	286	161	562	562

**Figure 6:**
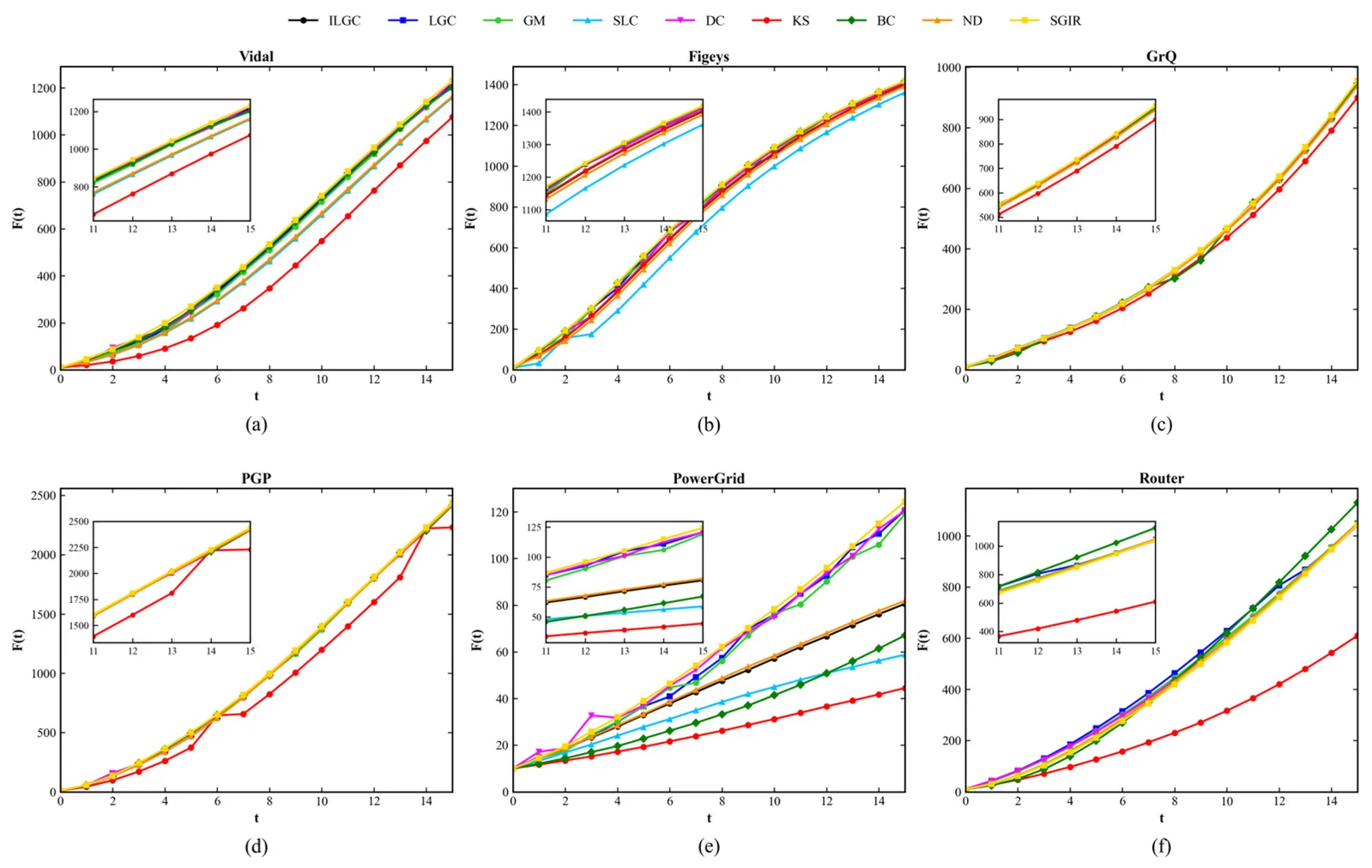
Spreading influence of the Top 10 ranking nodes of the SGIR and the baseline measures on 6 networks. The horizontal and vertical axes represent the time and number of infected nodes at time t. (**a**) Vidal; (**b**) Figeys; (**c**) GrQ; (**d**) PGP; (**e**) PowerGrid; (**f**) Router.

To further verify whether the Top 10 nodes uniquely identified by SGIR indeed exhibit stronger spiller characteristics in real spreading dynamics, we designed an additional simulation experiment for the above six networks. For each method, its Top 10 nodes are simultaneously set to the initial infected state (I), while all remaining nodes are set to the susceptible state (S). Subsequently, the SIR Model is used to simulate the transmission process, where t varies from 0 to 15, and each simulation is repeated 200 times independently. The cumulative count F(t) of infected nodes is averaged over all running results to obtain the final transmission curve. The results are shown in Figure 6.

The experimental results show that the SGIR algorithm exhibits propagation advantages in most networks. In Vidal, Figeys, and PGP networks, the propagation curve of SGIR was always at the top, and the final infection scale was significantly higher than that of other comparison algorithms.

SGIR is also firmly in the first echelon in PowerGrid and Router networks. Although ILGC shows strong propagation ability in the Router network, it is lower than SGIR in most networks.

### 5.7. Performance Evaluation Using Recognition Rate $R_{p}$

**Figure 7:**
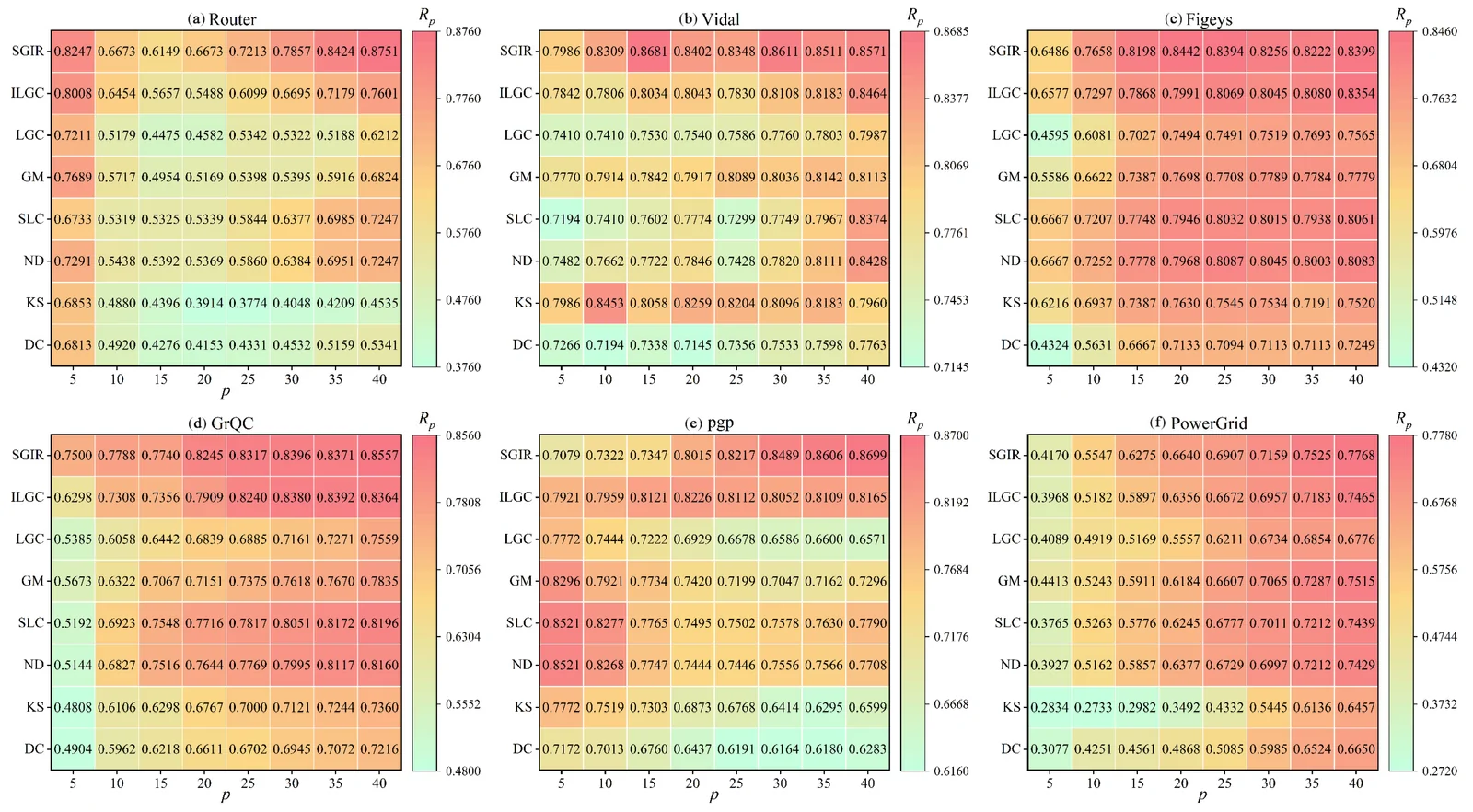
Comparison of recognition rates $R_{p}$ of different centrality methods on six real networks. Each subfigure shows the matching degree between the set of critical nodes identified by each method and the set of true influential nodes of the SIR Model as the propagation probability p changes. (**a**) Router; (**b**) Vidal; (**c**) Figeys; (**d**) GrQC; (**e**) pgp; (**f**) PowerGrid.

In order to further verify the effectiveness of the proposed SGIR method in the critical node identification task, the recognition rate $R_{p}$ is used as the evaluation index to compare the high-influence node sets screened by different methods with the real critical node sets obtained based on the SIR Model. The higher the recognition rate $R_{p}$, the higher the coincidence degree between the nodes identified by the algorithm and the nodes with strong real propagation ability, that is, the method has a more accurate ability to locate key nodes. The experiments were carried out on six real networks including pgp, Figeys, GrQC, PowerGrid, and Router, and the propagation probability parameter p was changed to analyze the recognition performance of different methods under different propagation conditions. The experimental results are shown in Figure 7.

In the PGP network, the $R_{p}$ value of SGIR increased from 0.8015 to 0.8699, significantly outperforming single local structure methods such as DC and KS, indicating that it can effectively utilize multi-order neighborhood information to locate core nodes. In the Figeyes network, the $R_{p}$ value of SGIR remained stable (0.8442–0.8399), while methods like LGC and ILGC fluctuated under specific propagation probabilities. SGIR achieved more stable evaluation by integrating semi-local structure and K-shell global influence. In the GrQC network, SGIR’s $R_{p}$ continued to increase to 0.8557, benefiting from its consideration of both the node’s own connection ability and high-order neighborhood correlation.

For the sparse PowerGrid network, SGIR’s $R_{p}$ reached 0.7768 under high propagation probabilities, outperforming most comparison algorithms, proving that global influence calculation can effectively compensate for the lack of local information. In the Vidal network, SGIR achieved the highest $R_{p}$ (approximately 0.78 to 0.86) in almost all propagation probabilities. The modular structure of this network is obvious, and the bridging nodes connecting different functional modules are crucial for propagation. SGIR integrates semi-local embedding features and global position information to stably identify such nodes, while methods like degree centrality or betweenness centrality are difficult to effectively discover such locally insignificant but structurally significant nodes. In the Router network, SGIR’s $R_{p}$ remained at a high identification level, demonstrating good robustness.

Overall, SGIR achieved a high critical node matching rate in networks of different scales and structures. Compared with methods like DC, SLC, ND, KS, GM, LGC, and ILGC, by jointly modeling semi-local structure information and global propagation influence, SGIR effectively improved the accuracy, stability, and generalization ability of critical node identification.

### 5.8. Time Complexity Analysis

The proposed SGIR method mainly includes three stages: semi-local influence calculation, global influence calculation, and comprehensive influence ranking. In the phase of semi-local influence calculation, the network adjacency matrix is constructed, and the second-order adjacency matrix $A^{2}$ and third-order adjacency matrix $A^{3}$ are calculated. Due to the sparse matrix storage, the time complexity of matrix multiplication in sparse complex networks is about O(m<k>) and $O(m<k{>}^{2})$, where <k> is the average degree of the network.

Then, the structural incidence matrix is constructed, and the structural energy offset and normalized semi-local influence of nodes are calculated, which only involves the traversal of non-zero elements of the matrix, so the time complexity is not more than O(m<k>). Therefore, the overall time complexity of the semi-local influence calculation phase is $O(m<k{>}^{2})$. In the global influence calculation phase, the K-shell decomposition is used to obtain the core number of each node, whose time complexity is O(m+n). Then, the adaptive propagation radius is determined according to the average shortest path length of the largest connected subgraph, and the process is executed only once. For each node, a breadth-first search (BFS) is performed within the propagation radius r, which ranges the propagation radius as 3≤r≤5 and hence r is constant. Each search visits only the local neighborhood of the node, and its average time complexity is about $O(<k{>}^{r})$. After traversing all nodes, the time complexity of the global influence calculation phase is $O(n<k{>}^{r})$. Finally, the semi-local influence and the global influence were linearly fused to obtain the comprehensive influence of nodes, and the nodes were sorted according to the influence size, whose time complexities were O(n) and O(nlogn), respectively. The overall time complexity of the SGIR algorithm can be expressed as $O(m<k{>}^{2}+n<k{>}^{r}+n\log n)$. Since the propagation radius r is a constant at most 5, $n<k{>}^{r}$ is a linear growth term with respect to the number of nodes. In actual complex networks, m=O(n) and <k><<n are usually satisfied, so the SGIR algorithm has good computational efficiency and scalability and can meet the needs of critical node identification tasks in large-scale complex networks.

**Figure 8:**
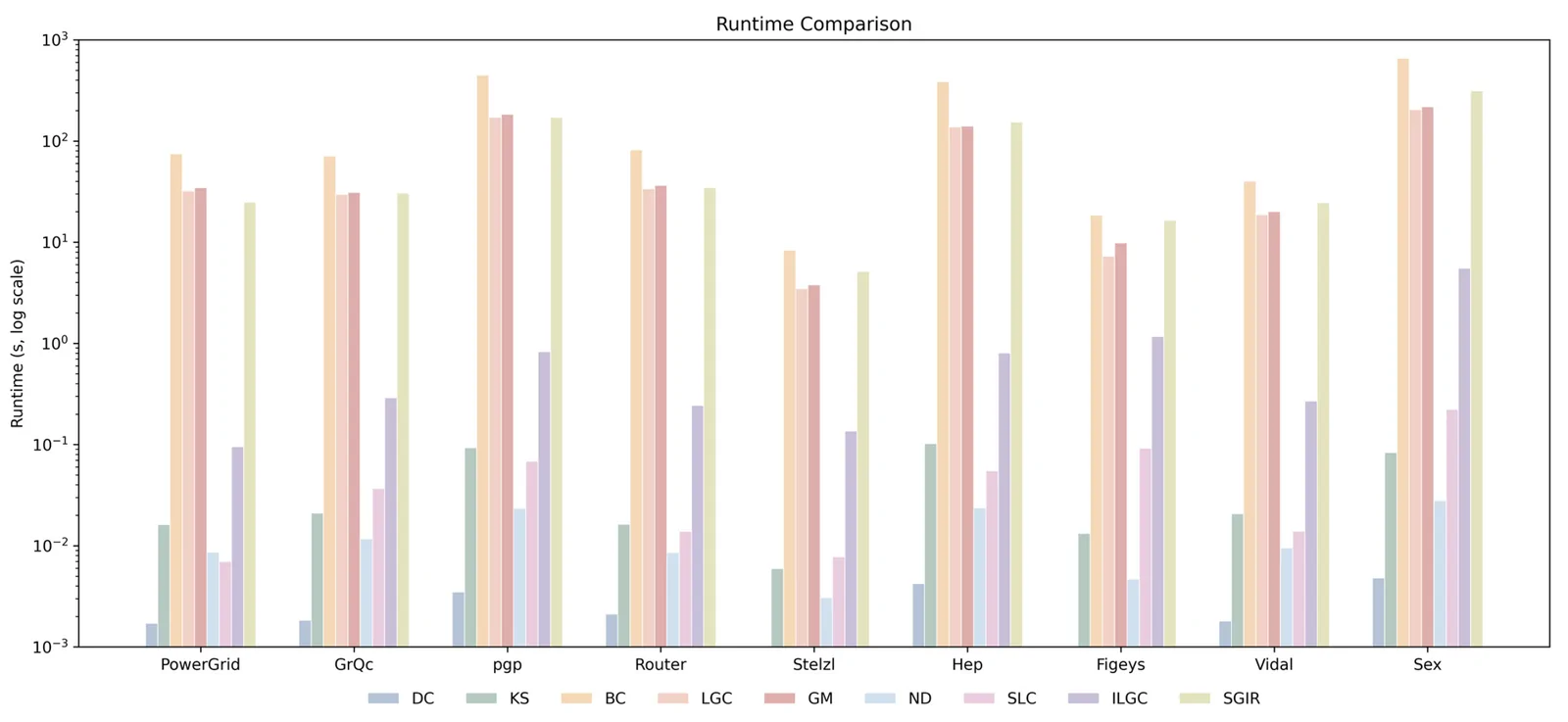
The running time of SGIR is compared with eight compared methods on nine real networks.

To further investigate the additional computational overhead brought by high-order sparse matrix multiplication for constructing structural correlation matrices, we conduct running time experiments on nine real networks to compare the average running time of SGIR with eight other comparison methods DC, KS, BC, ND, SLC, GM, LGC, and ILGC. The experimental results are shown in Figure 8.

According to the figure, the local metrics DC, ND, and SLC are the fastest because they only need to traverse the direct neighborhood of each node. KS is slightly slower, but still in the same order of magnitude.

In contrast, BC, LGC, and GM are the costliest: BC takes the longest time to run in each network; LGC and GM are slightly lower but equally expensive. ILGC restricts the accumulation to a bounded neighborhood and is thus significantly faster than LGC and GM but sacrifices part of the global information. SGIR is in the middle: slower than DC, KS, ND, SLC, and ILGC but close to LGC and GM and significantly faster than BC. In addition, the running time of SGIR grows nearly linearly with the network size, instead of rising sharply like BC, LGC, and GM, which is consistent with the complexity based on sparse matrices in theoretical analysis. This indicates that although the introduction of higher-order structural information increases the computational cost, the sparse matrix operation keeps the extra overhead within a practical near-linear range, and SGIR is still suitable for large-scale complex networks.

### 5.9. Sensitivity Analysis of the Maximum Radius

To test whether a fixed upper bound limits the ability of the global influence model to capture the complete propagation structure of networks with unusually large or small diameter, Kendall’s $\tau_{b}$ evaluation was calculated while relaxing the upper bound to $r_{\max}=6$ and $r_{\max}=7$, holding all other components of SGIR constant. Table 6 reports Kendall’s *τ_b_* correlations between the SGIR rankings obtained at $r_{\max}=$ 5, 6, and 7 and the SIR-based reference rankings for all nine networks.

The results show that relaxing the upper limit from 5 to 7 has little effect on Kendall’s $\tau_{b}$ value, and the five networks (Sex, Figeys, Vidal, GrQC, Stelzl) are basically unchanged, and the maximum deviation of the full dataset is only 0.011. These results show that $r_{\max}=5$ already captures basically all the global propagation information useful for ranking, which is consistent with the small-world property of real complex networks mentioned in Section 4.2. However, a larger cut-off value only increases the computational cost without the corresponding accuracy improvement.

## 6. Conclusions

In order to solve the problems of insufficient utilization of local structure information, inadequate description of global propagation ability and limited discrimination ability of nodes in the identification process of critical nodes in complex networks, this paper proposes a key node ranking method for complex networks combining semi-local and global influence, named SGIR. Firstly, in the semi-local influence calculation stage, the second-order neighborhood, the third-order neighborhood, and the triangular closed structure of the node were used to construct the structural correlation matrix, and the structural energy migration mechanism was introduced to describe the structural influence of the node in the local network from multiple perspectives. Secondly, in the global influence calculation stage, based on the K-shell decomposition, the distance attenuation mechanism is introduced to weighted aggregate the influence of core nodes within the limited propagation range, and the adaptive propagation radius is designed by combining the average shortest path length of the network, so that the global influence can more truly reflect the propagation ability of nodes in the network. Finally, the semi-local influence and global influence were fused to obtain the comprehensive influence ranking of nodes, which realized the collaborative modeling of local topology information and global structure information, and improved the accuracy and stability of critical node identification. In order to verify the effectiveness and robustness of the proposed SGIR method, systematic experiments are carried out on nine real complex networks from different fields such as communication network, power grid network, biological network, and social network. Experimental results show that the proposed SGIR method has good stability and generalization ability in different networks.

In the future, the SGIR method still has room for further research and improvement. On the one hand, it can be extended to weighted networks, directed networks, multi-layer networks, and dynamic time-varying networks to further enhance the adaptability of the algorithm to complex scenes. On the other hand, advanced technologies such as node attribute information, graph neural network and network representation learning can be combined to improve the ability of node influence modeling.

## Figures and Tables

**Table 1:** Basic information of the network of the dataset.

Network	*n*	*m*	*<k>*	*k*_max_	*C*	*d*	$\bar{L}$
Figeys	2239	6432	5.745	314	0.3995	0.0025	3.844
Vidal	3023	6149	4.1	129	0.0658	0.0026	4.840
GrQC	5242	14,484	5.52	81	0.5296	0.0011	6.049
Hep	9877	25,973	5.26	65	0.4714	0.0057	5.945
Stelzl	1702	3155	3.7	95	0.0060	0.0022	4.848
PowerGrid	4941	6594	2.7	19	0.0801	0.0005	18.989
Sex	16,730	39,044	4.7	305	0.0070	0.0008	4.204
pgp	10,680	24,316	4.554	205	0.2659	0.0004	7.486
Router	5022	6258	2.5	106	0.0116	0.0005	6.449

**Table 2:** Monotone values of the different methods for the nine networks. The highest value in each column is indicated by bold and the second highest value is underlined.

Network	DC	BC	SLC	KS	ND	GM	LGC	ILGC	SGIR
Sex	0.8057	0.9336	0.9935	0.7531	0.9937	**0.9999**	**0.9999**	0.9993	**0.9999**
Hep	0.7896	0.5834	0.9782	0.6907	0.9799	**0.9998**	**0.9998**	0.9975	**0.9998**
Figeys	0.597	0.6278	0.9886	0.5676	0.9887	0.9937	**0.9939**	0.9932	0.9937
Vidal	0.6667	0.744	0.9753	0.5655	0.9769	**0.9988**	**0.9988**	0.9962	**0.9988**
GrQ	0.7916	0.4849	0.9759	0.6924	0.9782	**0.9993**	**0.9993**	0.9969	**0.9993**
Stelzl	0.5527	0.6355	0.9677	0.4476	0.9699	0.9980	**0.9982**	0.9941	0.9979
Router	0.2886	0.3037	0.9126	0.0691	0.9199	**0.9969**	**0.9969**	0.9833	0.9964
pgp	0.6193	0.5115	0.9499	0.4805	0.9552	**0.9997**	**0.9997**	0.9938	**0.9997**
PowerGrid	0.5926	0.8317	0.8569	0.2459	0.8866	**0.9998**	**0.9998**	0.9726	**0.9998**

**Table 6:** Kendall’s $\tau_{b}$ of SGIR for different values of the maximum radius $r_{\max}$.

$r_{\max}$	Sex	Hep	Figeys	Vidal	GrQC	Stelzl	Router	PowerGrid	pgp
5	0.8603	0.8221	0.7043	0.8272	0.7855	0.9063	0.8214	0.6155	0.7954
6	0.8603	0.8246	0.7043	0.8272	0.7849	0.9063	0.8104	0.6192	0.7868
7	0.8603	0.8246	0.7043	0.8272	0.7849	0.9063	0.8104	0.6154	0.7856

## Data Availability

The original contributions presented in this study are included in the article. Further inquiries can be directed to the corresponding author.
